# Molecular docking, chemo-informatic properties, alpha-amylase, and lipase inhibition studies of benzodioxol derivatives

**DOI:** 10.1186/s13065-021-00766-x

**Published:** 2021-06-23

**Authors:** Mohammed Hawash, Nidal Jaradat, Suhaib Shekfeh, Murad Abualhasan, Ahmad M. Eid, Linda Issa

**Affiliations:** 1 Department of Pharmacy, Faculty of Medicine and Health Sciences, An-Najah National University, P.O. Box 7, Nablus, 00970 Palestine; 2 Chemometrics and Analytical Chemistry, Modern Testing Services, Povinostr. 52, 86153 Augsburg, Germany

**Keywords:** Benzodioxole, α-Amylase, Lipase, Acarbose, Bioactivity, Molecular docking

## Abstract

Currently, available therapies for diabetes could not achieve normal sugar values in a high percentage of treated patients. In this research project, a series of 17 benzodioxole derivatives were evaluated as antidiabetic agents; that belong to three different groups were evaluated against lipase and alpha-amylase (α-amylase) enzymes. The results showed that 14 compounds have potent inhibitory activities against α-amylase with IC_50_ values below 10 µg/ml. Among these compounds, **4f** was the most potent compound with an IC_50_ value of 1.11 µg/ml compared to the anti-glycemic agent acarbose (IC_50_ 6.47 µg/ml). On the contrary, these compounds showed weak or negligible activities against lipase enzyme. However, compound **6a** showed the best inhibitory anti-lipase activity with IC_50_ 44.1 µg/ml. Moreover, all the synthesized compounds were undergone Molinspiration calculation, and the result showed that all compounds obeyed Lipinski’s rule of five. Molecular docking studies were performed to illustrate the binding interactions between the benzodioxole derivatives and α-amylase enzyme pocket. Related to the obtained results it was clear that the carboxylic acid, benzodioxole ring, halogen or methoxy substituted aryl are important for the anti-amylase activities. The potent inhibitory results of some of the synthesized compounds suggest that these molecules should go further in vivo evaluation. It also suggests the benzodioxole derivatives as lead compounds for developing new drug candidates.

## Introduction

Diabetes mellitus (DM) is a chronic metabolic disorder causing hyperglycemia, with many other disturbances in metabolizing carbohydrates and proteins. Diabetes is caused by an absolute deficiency in insulin secretion (type 1 DM) or insulin action (type 2 DM) [1]. The worldwide incidence of diabetes is increasing every year. Based on information from the International Diabetes Federation the estimated number of people with diabetes reached 30 million͑ in 1985, 150 million in 2000, and 246 million in 2007͑. They also expects the number diabetic patients may hit 380 million by 2025 [2]. Α-amylase is defined as an enzyme, which catalyzes the biochemical pathway for the hydrolysis of starch (Latin amylum) into simple sugars. This enzyme is present in the saliva in the human body and in some other mammals. This enzyme starts the chemical process of digestion [3, 4]. While, α-D-glucosideglucohydrolase, α-1,4-glucosidase, α-glucoside hydrolase, α-d-glucosidase, glucosidoinvertase, α-glucopyranosidase, maltase-glucoamylase, glucosidosucrase, glucoinvertase, and maltase are α-glucosidase enzymes which found on the border of the small intestine and act upon hydrolysis of α-(1,4) bonds between monosaccharide units. In contrast to β-glucosidase, α-glucosidase hydrolyzes starch and disaccharides to glucose. Maltase seems to be a similar enzyme that cleaves maltose, which is almost functionally equivalent [5, 6].

One of the recent widespread health problems is obesity. The increased body fat may have severe adverse effects on human health. Obesity is considered as one of the leading causes of cancer disease according to the WHO reports [7]. In general, a person is characterized as obese when his measured body mass index (BMI), is more than 30 kg/m^2^, while between 25 and 30 kg/m^2^ is classified as overweight [8]. Obesity occured due to several factors starting with high food intake and low physical training, and it may depend on genetic susceptibility [8, 9]. A few cases have appeared primarily due to genes, endocrine disturbances, some ingested medications, or mentally related problems [10]. The relationship between obesity and diabetes is made by a progressive defect or decrease in insulin secretion as well as coupled with a progressive increase in insulin resistance. Both insulin resistance and insulin deficiency appear very prematurely in obese patients, and both worsen similarly towards diabetes [2, 11].

**Fig. 1:**
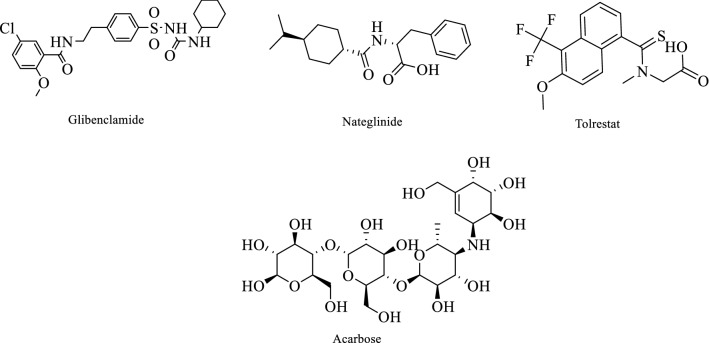
Structures of antidiabetic drugs

The 1,3-benzodioxol core structure present in the safrole compound, could be isolated from *Crocus sativus* L. and *Ocotea pretiosa* Mez plants, and interesting biological activities. The benzodioxole nucleus has been utilized in drug discovery for the synthesis of novel molecules with various biological activities, including anti-schistosomicidal, antiepileptic, analgesic, antituberculosis, and antimicrobial potentials [12–18]. On the other side, many structures containing carboxylic acid (COOH) group such as Nateglinide and tolrestat (Fig. 1) and aryl-amide group Glibenclamide (Fig. 1) were used as antidiabetic drugs, and these functional group considered as essential functional groups for the activities. Acarbose, which is used for diabetes disease, has polyphenol groups in its structure [19] (Fig. 1).

**Scheme 1:**
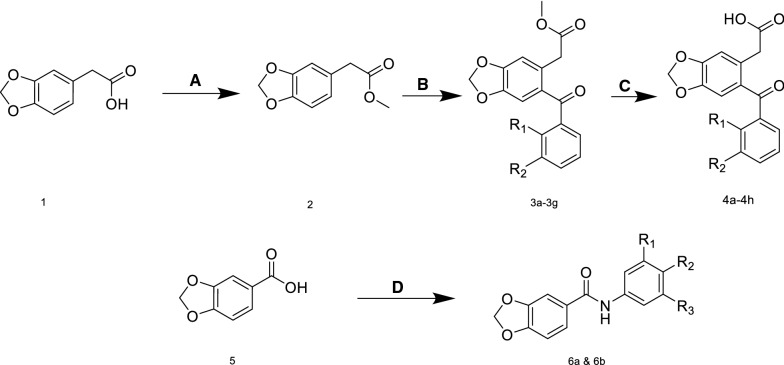
The reaction steps A) methanol, oxalyl chloride B) DCM, P2O5, aryl-carboxylic acid, C) MeOH/THF/H2O, LiOH reflux, D) Aniline derivatives, DCM, EDC, DMAP. * (R groups were listed in Table 1).** (**1**: 2-(benzo[d][1,3]dioxol-5-yl)acetic acid, **2**: methyl 2-(benzo[d][1,3]dioxol-5-yl)acetate, **5:** benzo[d][1,3]dioxole-5-carboxylic acid)

**Fig. 2:**
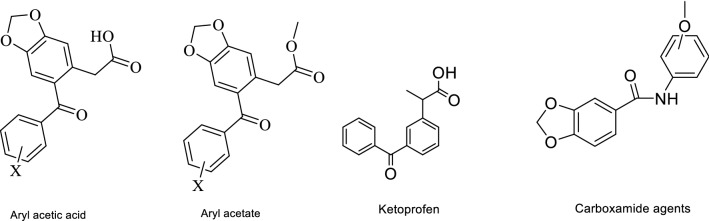
Structures of benzodioxole structures and ketoprofen

Benzodioxole derivatives were synthesized previously by our team as illustrated in Scheme 1. These compounds were designed and synthesized previously to be COX enzyme inhibitor candidates [20]. However, the main pharmacophore features of COX inhibitors Non-steroidal anti-inflammatory drugs (NSAIDs) were identified as; (i) aryl acetic acid (ionic center and first hydrophobic region), (ii) Second non-coplanar halogenated aryl ring (the second region lies under and out of the plane with the first hydrophobic area) and (iii) non-rotatable bond between the aromatic rings [19]. The first biological target was the COX enzymes, and the compounds with aryl acetic acids and aryl acetate (Fig. 2) were showed moderate to potent activities as COX inhibitors, and their selectivities were better than ketoprofen NSAID [20], while the benzodioxole-carboxamide derivatives (Fig. 2) were synthesized with poly-methoxyphenyl moiety as anticancer agents but their cytotoxic activities were not very potent [21].

Regarding the similarities in the structure between the antidiabetic agents (Fig. 1) and the evaluated compounds (Fig. 2) especially the presence of COOH, aryl or amide, and halogen-substituted on the aryl rings, the idea came to evaluate these compounds on the second target regarding α-amylase enzyme, and as it is common scientifically a lot of structures may have synthesized for a specific target and later they may have shown activities on other biological targets. In this study, the three classes benzodioxole namely: benzodioxole aryl acetate (**3a–3 g**), benzodioxole aryl acetic acid (**4a–4 h**), benzodioxole-carboxamide (**6a & 6b**) were assayed for their anti-amylase and anti-lipase activity.

## Results and discussion

### Antidiabetic activity

The in vitro antidiabetic activity of the synthesized compounds was investigated by assessing their α-amylase inhibitory effects. Acarbose was used as a positive control. The inhibition power of a serial of five concentrations in the range of 1, 10, 50, 100, and 500 µg/ml were used to calculate the IC_50_ values of these compounds (Table 1).

**Table 1:** All chemical structures of evaluated compounds and their IC_50_ values regarding α-amylase and lipase inhibitory activities

Structure	Compound code	R_1_	R_2_	R_3_	IC_50_ (µg/ml)	
					α-amylase	Lipase
	**3a**	Cl	H	H	2.52 ± 1.1	145.49 ± 2.5
	**3b**	H	H	H	61.45 ± 1.5	> 400
	**3c**	I	H	H	39.85 ± 1.3	> 400
	**3d**	H	I	H	16.36 ± 0.75	118.86 ± 2.1
	**3e**	Br	H	H	2.57 ± 0.90	78.37 ± 1.72
	**3f**	H	Br	H	9.29 ± 1.4	> 400
	**3 g**	Cl	H	Cl	3.94 ± 2.01	> 400
	**4a**	Cl	H	H	8.88 ± 2.10	> 400
	**4b**	H	Cl	H	1.25 ± 1.01	> 400
	**4c**	H	H	H	4.12 ± 1.63	213.82 ± 2.1
	**4d**	I	H	H	7.95 ± 1.15	187.7 ± 1.23
	**4e**	H	I	H	3.85 ± 0.98	201.2 ± 2.37
	**4f**	Br	H	H	1.11 ± 1.07	290.7 ± 2.23
	**4 g**	H	Br	H	4.90 ± 2.21	225.8 ± 1.43
	**4 h**	Cl	H	Cl	9.50 ± 0.56	206.6 ± 1.96
	**6a**	O–CH_3_	O–CH_3_	H	8.547 ± 1.55	44.47 ± 2.30
	**6b**	O–CH_3_	H	O–CH_3_	4.286 ± 1.01	100.8 ± 1.73
Acarbose					6.47 ± 2.01	-
Orlistat					–	25.01 ± 1.71

Table 1 shows a potent anti-amylase activity of benzodioxole derivatives, and 14 compounds with potent activities (IC_50_ < 10 µg/ml), and 3 compounds with moderate activities (IC_50_ 16–61 µg/ml). The compounds with acetic acid **4a–4 h** showed better activity range than the aryl acetate **3a–3 g**. and amide group **6a–6b.** The most two active compounds were **4b** and **4f** from the aryl-acetic acid class, with IC_50_ 1.25, and 1.11 µg/ml respectively (five to six folds more potent than acarbose). The Ideal halogen on the aryl should be Cl or Br atoms for better activity, but with larger atom-like I the activity was decreased especially in the aryl acetate class and compound **3c** was one of the worst active compounds with an IC_50_ value of 39 µg/ml. However, no clear relationship between the positions of the halogens on the phenyl ring and the activity. The carboxamide class showed moderate activities against α-amylase with IC_50_ 4.28 and 8.54 µg/ml for **6b** and **6a** compounds, respectively. While the lipase inhibition activity for compounds was weak or negligible and the most active compound was **6a** with IC_50_ 44.47 µg/ml**,** in comparison with orlistat positive control with IC_50_ values 25.01 µg/ml.

### Lipinski’s rule (RO5) of evaluated compounds

A library of 17 compounds was synthesized previously, and their chemical properties were calculated accordingly. Results showed that tested compounds have better-predicted value of molecular weight (M.Wt.) (g/mol), hydrogen bond acceptor (HBA) and donor (HBD), logP, and polar surface area (PSA) (A^2^). Moreover, Lipinski’s rule of five (RO5) analysis presents that all compounds, obey the RO5 rule and possess good comparable values against standard M.Wt. (< 500 g/mol) (range 258.34–424.19 g/mol), HBA (< 10) (range of compounds 2–6), HBD (< 5) (range 0–1) and logP (< 5) values (2.64–4.54) [22]. Furthermore, the polar surface area (PSA) of compounds is defined as the surface sum over all polar atoms, primarily oxygen and nitrogen, also including their attached hydrogen atoms. The polar surface area (PSA) parameter is usually used for the drug's optimization ability to permeate cell membrane. Previous research data showed the standard value of PSA should be less than 89 A^2^ [23]. Our predicted results showed that the evaluated compounds possessed less than standard values PSA results within the range 26.30–72.84 A^2^. The number of rotatable bonds (nRotb) is also considered as a very good parameter to descript the oral bioavailability of chemicals, and it was chemically defined as any single non-ring bond, bounded to non-terminal heavy atoms. A previous research data showed that nRotb in chemical compounds must be less than 10 which prove the high possibility of good oral bioavailability in rat [24]. Our predicted results showed that all evaluated compounds were within a standard range which showed their good oral bioavailability character (range 4–6).

### Docking studies

Previously, some natural products have been reported as inhibitors of human pancreatic α-amylase (HPA); mainly saccharides such as Acarbose [25], or flavonoid derivatives such as myricetin and montbretin A (MbA); which is glycosylated flavonol attached to a caffeic acid moiety [26]. The crystal structure of MbA bound to HPA has been determined (PDB id: 4w93, with resolution 1.35 Å), showing the dominance of hydrogen bond interactions between HPA residues and the hydroxyl groups of MbA. Interestingly, the x-ray structure showed that the bound conformation of MbA is characterized by intermolecular pi-pi stacking between the two aromatic rings (the aromatic ring A of the flavonoid core and the aromatic ring of the caffeic moiety). The hydrogen bonds are either formed directly or water-mediated between the bound ligand and the protein residues; such as the catalytic residues E233 and D197, besides other residues I235, H201, H305, K200, E240, H101, Q63, N298, D356, R159, and D300 moiety [26]. Similarly, Acarbose showed hydrogen bond interactions with (PDB id 1OSE) similar residues of Pig Pancreatic α-amylase; D197, E233, H101, H201, K200, Y62, Y151, and others [27]. Other publication has already established a pair of residues; an aspartate (as nucleophile) and glutamate (as acid–base), as critical catalytic residues for the α-amylase function [28].

To understand the interactions between our evaluated benzodioxole derivatives with the binding pocket of the α-amylase enzyme, the most active compound from each group were selected (**3a**, **4f**, and **6b**) to investigate their possible docking solution inside the structure of the α-amylase enzyme (PDB 4w93) using free-source docking software rDock. This software depends on a combination of stochastic and deterministic search methods: a three-stage genetic algorithm followed by Monte Carlo simulation and simplex minimization [29]. In order to generate low energy, ligand poses inside the binding cavity defined to be the residues around the bound ligand (MbA) within a distance of 6 Å. The suggested docking solutions were visually examined for their interactions with the critical residues mentioned above.

**Fig. 3:**
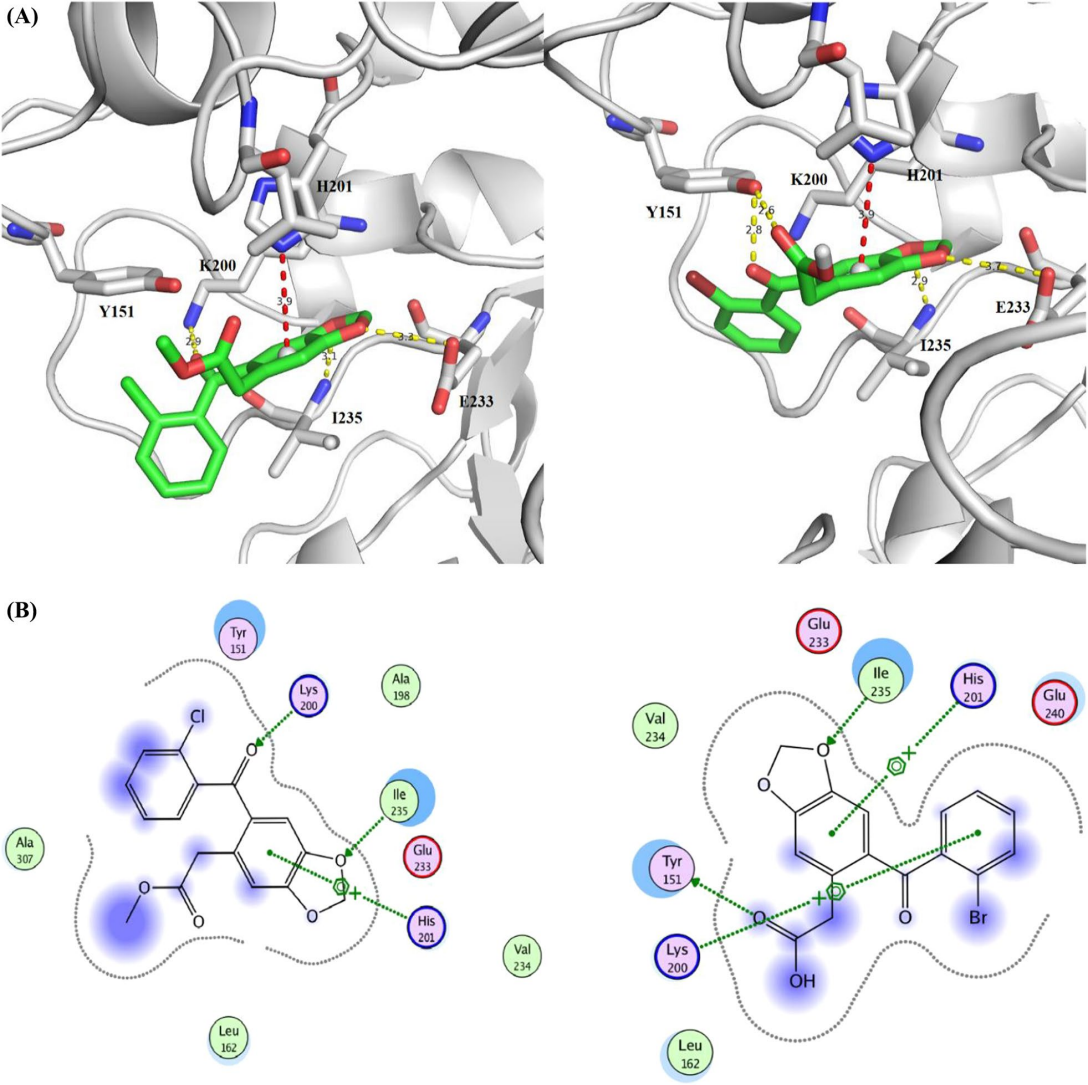
**A** Docking Solution of the compound **3a** (left) and **4f** (right) inside the binding pocket of alpha-amylase (pdb code: **4w93**) forming hydrogen bonds with the residues I235 and E233; while making and charge-π interactions with H201. Other residue like Y151 is also able to make hydrogen bonds or hydrophobic interactions. **B** The interaction map of the compound **3a** (left) and **4f** (right) inside the binding pocket of human pancreatic alpha-amylase (pdb code: **4w93**) forming hydrogen bonds with the residue I235; while making and charge-π interactions with H201, and other possible HB and hydrophobic interactions with the surrounding residues

**Fig. 4:**
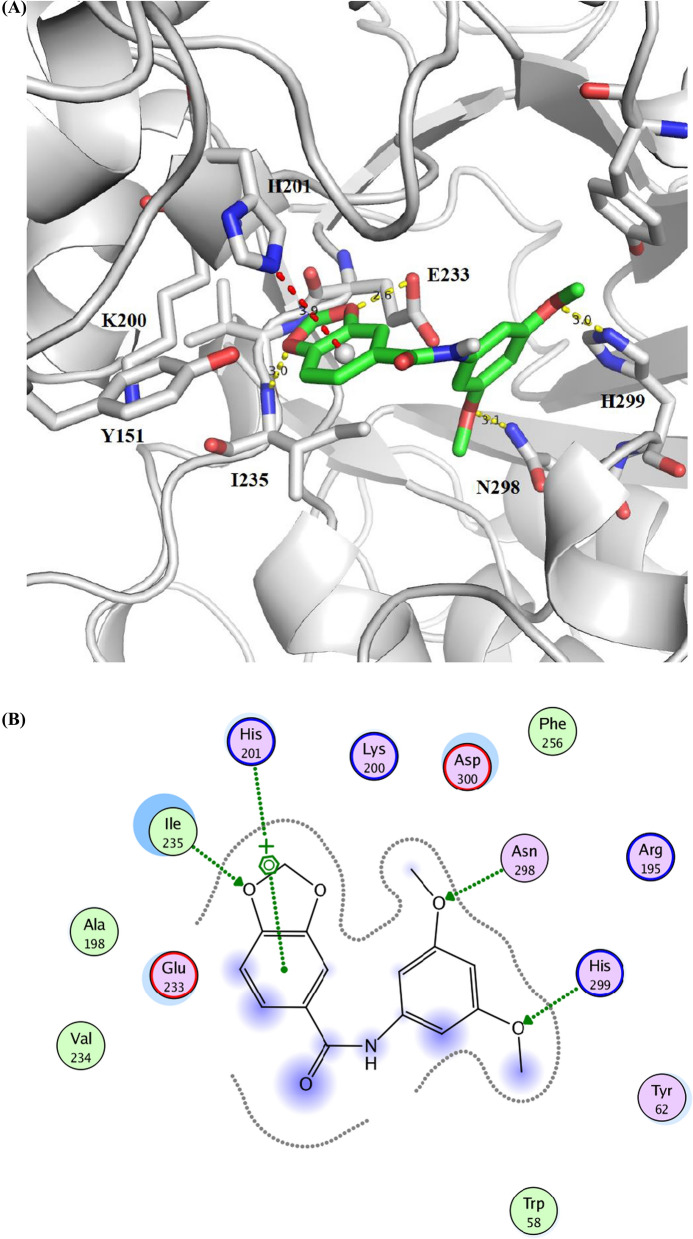
**A** Docking Solution of the compound **6b** inside the binding pocket of alpha-amylase (pdb code: **4w93**) in the proximity of residues I235 and E233; forming additional charge-π interactions with H201. Other residues like N298, H299, Y62, and Y151 could also form hydrogen bonds or π-π interactions with the bound inhibitor. **B)** The interaction map of the compound **6b** inside the binding pocket of human pancreatic alpha-amylase (pdb code: **4w93**) forming hydrogen bonds with the residue I235; while making and charge-π interactions with H201, and other possible HB and hydrophobic interactions with the surrounding residues; H299 and N298

Both compounds **3a** and **4f** could be fitted inside the binding pocket of HPA in the proximity to residues I235 and E233, which suggests possible direct or water-mediated hydrogen bonds with the oxygen atoms of the dioxol ring; while the fused benzene ring can form a charge-π Interaction with the histidine residue H201. The attached aryl can form hydrogen bonds and π–π stacking with residues like K200 and Y151, respectively (Fig. 3). On the other hand, compound **6b** was docked inside the α-amylase binding pocket showing also possible hydrogen bonds with the residues I235, E233 besides charge-π interactions with H201; stabilized by interactions with other surrounding residues like H299 and N298 (Fig. 4). Unlike the binding of Acarbose and montbretin A; which depends mainly on hydrogen bonds, these docking results suggest that the driving force of the binding for benzodioxole derivatives are a mixture of hydrogen bonds, charge- π, and hydrophobic π–π stacking.

## SAR results

The results of both *in-vitro* α-amylase inhibitory activities of the evaluated compounds shown in Table 1 and the docking studies with interaction map (Fig. 4), illustrate that compounds with a chemical feature of benzodioxole aryl acetic acid group (**4a-4 h**) have more potent activities compared to those having a benzodioxole aryl acetate group (**3a-3 g**), the average of IC_50_ values were 5.19 and 19.42 µg/ml respectively. The main difference between these two groups was the −COOH versus −COOCH_3_, and and the related interaction map (Fig. 4). The difference in activity could be explained by the Hydrogen bond interactions between the COOH and Tyr151, which was absent in the benzodioxole aryl acetate group (**3a–3 g**). However, in the Carboxamide group **6a–6b**; eventhough neither COOH nor COOCH_3_ groups were present, but still they showed potent activities with low IC_50_ values. This potent activity could be explained by the molecular docking studies showing new binding interactions between the Methoxy groups that are attached on the phenyl ring with Asn 298 and His 299 amino acids. This new interaction may compensate the absence of acrboxylated hydrogen interaction of the prevois groups. It was clear that the benzodioxole ring in all compounds made strong binding interactions with Ile235 and His201, which indicated that this moiety is an important pharmacophore for the activities. In the first group (**3a–3 g**) the presence of halogen atom is important for the activities especially with atoms smaller than I such as Br or Cl. Compounds without halogen (**3b**) has the lowest activity and compounds with I (**3c–3d**) have moderate activities. The bioactivity results illustrate that the position of Halogen atoms has no effect on the compound potency.

### Conclusion

The evaluated compounds showed potent activity against the α-amylase enzyme, on the contrary they showed weak or negligible activities against lipase enzyme. The Molinspiration cheminformatics calculation showed that these compounds are possible candidates to be an oral drug because they obey the Lipinski role of five. The docking studies suggest potent inhibition of α-amylase is derived from strong interactions with the catalytic residue E233 and the neighbor residue H201, besides other stabilizing interactions with surrounding residues. However, the in-vitro results showed potent antidiabetic candidates with activity better or very close to the positive control Acarbose. The main pharmacophore features of the compounds was identified as carboxylic acid, benzodioxole ring, Halogen atom-like Br or Cl, as well as the presence of methoxy group, will enhance the activities. These results encourage other researchers to complete studies of those compounds as well as encourage us to design more benzodioxole derivatives with methoxy aryl acetic acid features and encourage us to make in-vivo studies in the future.

## Experimental section

### Materials

The following materials were purchased from Frutarom (UK): β-carotene, porcine pancreatic amylase enzyme solution, starch, dinitrosalicylic acid (DNSA), methanol, trolox, gallic acid, Na_2_CO_3_, and acarbose.

All the tested compounds were synthesized previously by our team. The compounds are classified into three different groups: benzodioxole aryl acetate (**3a–3 g**), benzodioxole aryl acetic acid (**4a–4 h**) [20], benzodioxole-carboxamide (**6a & 6b**) [21], see Table 1.

### α-Amylase inhibitory activity method

The α-amylase inhibitory assessment was based on the Wickramaratne et al. protocol [30] with some slight alterations in some steps. The experimental part was carried out by following the 3,5-dinitrosalicylic acid (DNSA) procedure. Solutions of 20 mm sodium phosphate monobasic and sodium phosphate dibasic buffer involving 6.7 mm sodium chloride (NaH_2_PO_4_ and Na_2_HPO_4_, both including 6.7 mm NaCl, pH 6.9) were constructed by partially filling the beaker with NaH_2_PO_4_ and NaCl solution; the mixture was subjected to a magnetic stirrer. In contrast, the pH was adjusted by inserting a calibrated pH electrode in the solution. Then, the Na_2_HPO_4_ and NaCl solution was gradually added until the pH reached 6.9. A weight of 5.36 g of 20 mm Na_2_HPO_4._ 7H_2_O and 0.39 g of 6.7 mm NaCl were dispersed in distilled water to make 1 L and a weight of 2.76 g of NaH_2_PO_4_. H_2_O and 0.39 g of NaCl dissolved in distilled water to make 1 L. The stock solution for the synthesized molecules had a concentration of 1 mg/ml and was put in a minimum amount of 10% DMSO (1:100 dilution) and was then dispersed in a buffer of Na_2_HPO_4_/NaH_2_PO_4_ (0.02 M) and NaCl (0.006 M) at adjusted pH 6.9. Working solutions with concentrations of 1, 10, 50, 100 and 500 µg/ml were obtained by mixing 0.01, 0.1, 0.5, 1, and 5 ml of our synthesized molecules, respectively, and then diluting them with a buffer of Na_2_HPO_4_/NaH_2_PO_4_ (0.02 M) and NaCl (0.006 M) at pH 6.9 and then brined up to 10 ml using VF (10 ml). The acarbose was considered a reference and was established following the same previous steps used for the synthesized molecules.

A solution of α-amylase was (2 units/ml) was produced by dissolving 12.5 mg of amylase in a minimum amount of DMSO10%, which was then brined up to 100 ml with the previous phosphate buffer in a volumetric flask (V.F) (100 ml). A starch solution with a concentration of 1% (w/v) was prepared by suspending 1000 mg of starch in 100 ml of distilled water using V.F (100 ml), and then it was kept in a water bath at 37 °C until use, with occasional mixing to prevent starch precipitation. DNSA was used as a reactive reagent to react with reducing sugars to produce 3-amino-5- nitro salicylic acid that is highly absorbent of light at about 540 nm. It was prepared by dissolving 12 g of sodium potassium tartrate tetrahydrate in 8.0 ml of 2 M NaOH (8 g in 100 ml distill. water) then further dissolved in 20 ml of 96 mm of 3.5-dinitrosalicylic acid solution.

Then, 200 μl of the amylase solution (2 units/ml) was gently shaken with 200 μl of each of the VOs established working solutions, and then this was incubated at 37 °C for 10 min. Then 200 μl of the starch solution was added to each test tube, and there was further incubation for 3 min at 37 °C. The addition of 200 μl DNSA terminated the reaction and then boiling for 10 min at 85 to 90 °C. The mixture was then cooled to room temperature and diluted with 5 ml of distilled water, and the absorbance was recorded at 540 nm using a UV–Visible spectrophotometer. Replacement of the synthesized compounds with 200 μl of buffer was established to obtain the blank sample. In this protocol, acarbose was the positive control sample. The enzyme inhibitory activity was expressed as percent inhibition, and the following equation was used to determine the IC_50_ value for the tested compounds [31].

$$\alpha-{\text{amylase inhibitory }}\left( \% \right){\text{ }} = {\text{ }}\left[ {{\text{ABSblank }}{-}{\text{ ABStest}}} \right]/\left[ {{\text{ ABSblank}}} \right]){\text{ }}*{\text{1}}00\%$$

### Anti-lipase activity

A solution of 1 mg/ml of the synthesized compounds was mixed with 10% dimethyl sulfoxide (DMSO) and then diluted with 10% DMSO to produce five dilutions (40, 100, 200, 300, and 400 µg/ml). Orlistat was considered a reference in this inhibition protocol for pancreatic lipase and was then tested following the same steps used previously.

A freshly prepared stock solution of pancreatic lipase enzyme was established by suspending this enzyme in 10% DMSO to form 1 mg/ml. Firstly, 25 mg of lipase was suspended in a small amount of 10% DMSO, bringing the volume up to 25 ml in V.F (25 ml), this was then put in a water bath sonicator at 37 °C for 15 min. The stock solution of PNPB was constructed depending on the manufacturing structure (20.9 mg was obtained from PNPB and dispersed in 2 ml of acetonitrile) by dissolving 104.5 mg of PNPB in acetonitrile brined up to the volume of 10 ml in V.F (10 ml). The pancreatic lipase inhibition assay was conducted by adopting the procedure in the references with slight modifications [32–34]. From each working solution of the synthesized compounds prepared above, 200 µl of the synthesized compounds were taken and put in a separate test tube, then 100 µl of porcine pancreatic lipase (1 mg/ml) was added to it. The resulting mixture was then adjusted to 1000 µl after addition to the 700 µl of Tris–HCl solution, and then it was incubated in a water bath at 37 °C for 15 min. After the incubation, 100 µl of PNPB (*p*-nitrophenyl butyrate) solution was added to each test tube. This mixture was then incubated again in a water bath at 37 °C for 30 min. A solution characterized as a negative control was constructed without the synthesized compounds, using 100 µl of porcine pancreatic lipase (1 mg/ml) solution mixed with the Tris–HCl solution up to 1 ml after the addition of 900 µl. The same procedure was adopted for orlistat, which was a positive control. A Tris–HCl buffer was used to zero UV–Vis spectrophotometer at 405 nm. The pancreatic lipase effect was reported by measuring the hydrolysis of *p*-nitrophenolate to* p*-nitrophenol at 405 nm using a UV–Vis spectrophotometer device. The lipase inhibition activity of the synthesized compounds, or orlistat as a reference, was identified by measuring the effect on the enzyme reaction rate after the addition of the synthesized compounds and then comparing it with the control. The % inhibition of the synthesized compounds was calculated by using the following equation:

$${\text{Inhibitory lipase }}\left( \% \right){\text{ }} = {\text{ }}\left[ {\left( {{\text{A}}_{{\text{b}}} - {\text{ A}}_{{\text{s}}} } \right)/{\text{A}}_{{\text{b}}} } \right]{\text{ }}*{\text{1}}00$$

### Chemo-informatics properties of the tested compounds

The Chemo-informatics properties of the synthesized compounds were evaluated based on chemo-informatics properties and the Lipinski rule of five (RO5). Multiple online servers such as Molinspiration (http://www.molinspiration.com/), and Molsoft (http://www.molsoft.com/) were employed to predict the molecular properties designed compounds [35].

### Molecular docking

Protein Data Bank structure of human pancreatic α-amylase (PDB code: 4w93) was downloaded from the RCSB repository. The protein structures were analyzed with PyMOL v1.8. The native ligand, non-proteins atoms and crystallographic waters were removed. Polar hydrogen atoms were added to the previous structure, and side-chain amides and imidazoles were protonated assuming a physiological pH using an H++ server. Docking studies were performed using the open-source program rDOCK (rdock.sourceforge.net); which is a development of RiboDock [36]. The cavity was defined using the native ligand of crystal structure 4w93 (a flavonol glycoside called Monotrobtin A) within 6 Å around it. The standard docking protocol in rDOCK was used, including 3 stages of Genetic Algorithm search (GA1, GA2, GA3), followed by low-temperature Monte Carlo (MC) and Simplex minimization (MIN) stages (rDock Reference Guide, August 2015). Three representatives of the synthesized inhibitors (**3a, 4f, and 6b**) were selected to be docked in the binding pocket of the prepared crystal structure using the empirical score function of rDOCK keeping 20 docking solutions for each inhibitor to be sorted by their binding scores and later visually analyzed for the interactions between the pocket’s residues and the inhibitors.

## Data Availability

The datasets used and/or analyzed during the current study available from the corresponding author on reasonable request.

## Abbreviations

DM: Diabetes mellitus
IC_50_: 50% Inhibition concentration
µg: Microgram
ml: Milliliter
M.Wt.: Molecular weight
g/mol: Gram per mole
HBA: Hydrogen bond acceptor
HBD: Hydrogen bond donor
PSA: Polar surface area
RO5: Lipinski’s rule of five
nrotb: Number of rotatable bonds
GPCR: G-protein coupled receptor
NaHCO_3_: Sodium bicarbonate
NaOH: Sodium hydroxide
DMSO: Dimethyl sulfoxide
VF: Volumetric flask
